# Supplementary material for: Recombinant Human Prolidase (rhPEPD) Induces Wound Healing in Experimental Model of Inflammation through Activation of EGFR Signalling in Fibroblasts
Source: Molecules. 2023 Jan 14;28(2):851. doi: 10.3390/molecules28020851 (PMC9867103; doi:10.3390/molecules28020851)
Supplement: Supplementary file 1 [file molecules-28-00851-s001.zip › molecules-2129695-supplementary.pdf]

Supplementary Material

Article

Recombinant Human Prolidase (rhPEPD) Induces Wound Healing in Experimental Model of Inflammation Through Activation of EGFR Signalling in Fibroblasts

Weronika Baszanowska [1], Magdalena Niziol [2], Ilona Oscilowska [1], Justyna Czyrko-Horczak[3], Wojciech Miltyk [2] and Jerzy Palka [1,*]

[1] Department of Medicinal Chemistry, Medical University of Bialystok, Kilinskiego 1, 15-089 Bialystok, Poland, phone: +48-85-748-5706; e-mail: w.baszanowska22@wp.pl (W.B.), ilona.zareba@gmail.com (I.O.), pal@umb.edu.pl (J.P.)

[2] Department of Analysis and Bioanalysis of Medicines, Medical University of Bialystok, Kilinskiego 1, 15-089 Bialystok, Poland, phone: +48-85-748-5845; e-mail: magdalena.misiura@umb.edu.pl (M.M.); wojciech.miltyk@umb.edu.pl (W.M.)

[3] Department of Structural Chemistry, Faculty of Chemistry, University of Bialystok, Ciolkowskiego 1K, Bialystok, 15-245 Poland, phone: 85-738-8062; e-mail: czyrko.justyna@gmail.com (J.C.H.)

[*] Correspondence: pal@umb.edu.pl; Tel.: +48-85-748-5706

Description of data: The blots and images are described in the result section.
Supplementary data analysis presented- representative blots from Western blotting on Figures 3A,B and 4A,B.

1. Representative blots from Western blotting analysis presented in Figure 3A.

1.1. EGFR expression

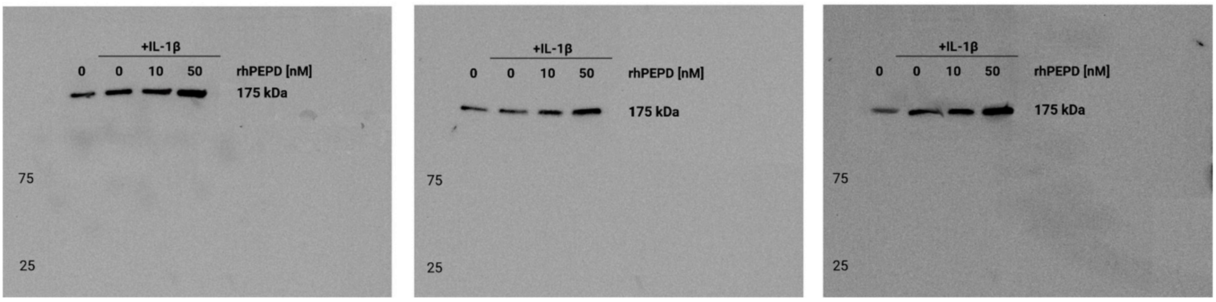

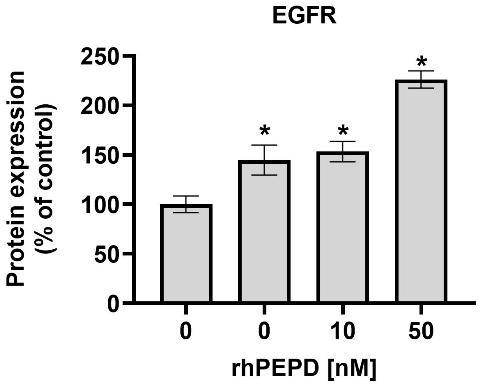

Figure S1. The EGFR expressions in rhPEPD-treated fibroblasts (rhPEPD, 10, 50 nM) for 24 h in presence or absence of IL-1β (10ng/ml). GAPDH expression was used as a loading control. The WB bands intensity of representative gels was quantified by densitometry and normalized to GAPDH. The densitometry values represent the mean (% of control) ± SD of three experiments. Statistical significances were expressed as *$P < 0.05$; vs. control cells (0 nM of PEPD, without IL-1β).

1.2. Phospho-EGFR expression

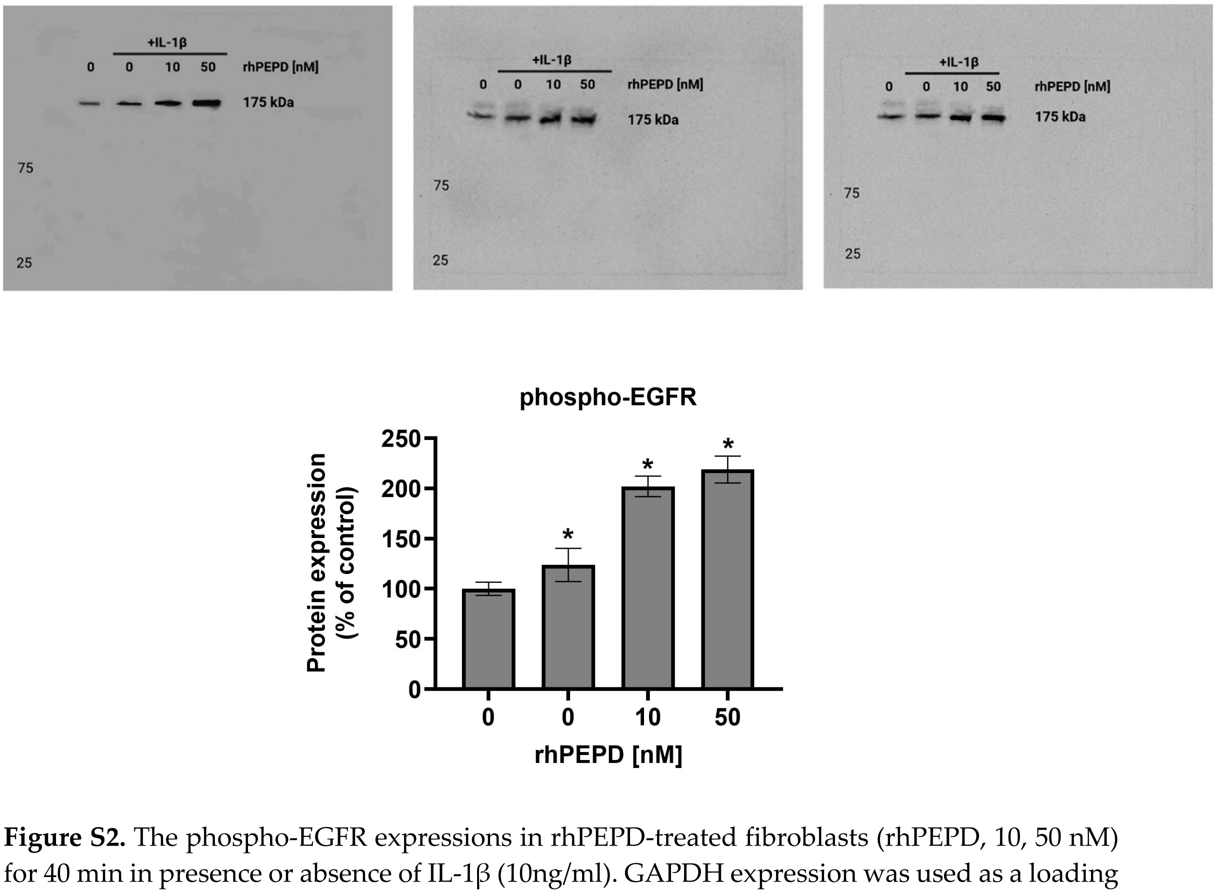

Figure S2. The phospho-EGFR expressions in rhPEPD-treated fibroblasts (rhPEPD, 10, 50 nM) for 40 min in presence or absence of IL-1β (10ng/ml). GAPDH expression was used as a loading control. The WB bands intensity of representative gels was quantified by densitometry and normalized to GAPDH. The densitometry values represent the mean (% of control) ± SD of three experiments. Statistical significances were expressed as *$P < 0.05$; vs. control cells (0 nM of PEPD, without IL-1β).

1.3. PI3K expression

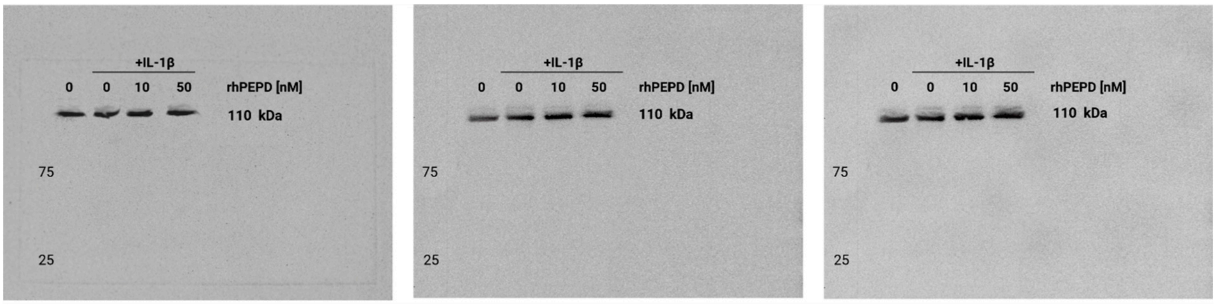

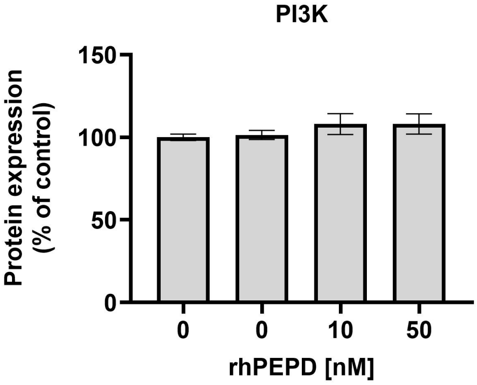

Figure S3. The PI3K expressions in rhPEPD-treated fibroblasts (rhPEPD, 10, 50 nM) for 24 h in presence or absence of IL-1β (10ng/ml). GAPDH expression was used as a loading control. The WB bands intensity of representative gels was quantified by densitometry and normalized to GAPDH. The densitometry values represent the mean (% of control) ± SD of three experiments. Statistical significances were expressed as *$P < 0.05$; vs. control cells (0 nM of PEPD, without IL-1β).

1.4. Phospho-PI3K expression

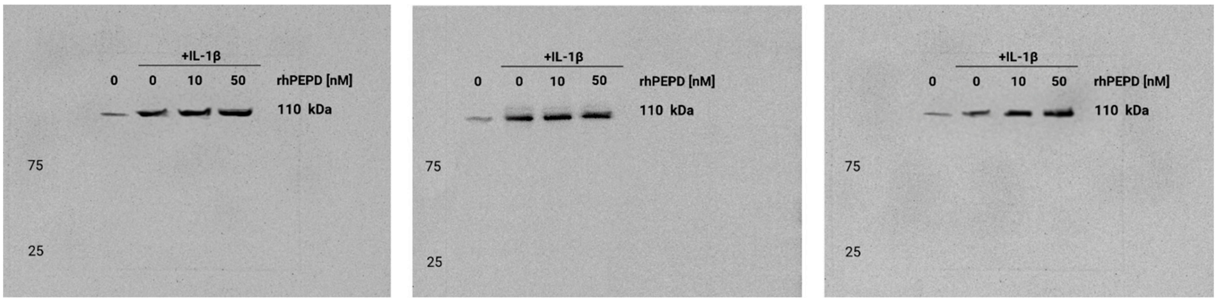

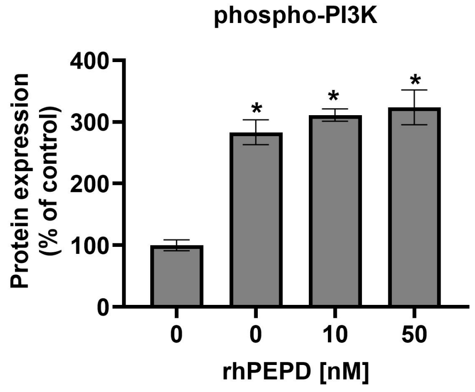

Figure S4. The phospho-PI3K expressions in rhPEPD-treated fibroblasts (rhPEPD, 10, 50 nM) for 40 min in presence or absence of IL-1β (10ng/ml). GAPDH expression was used as a loading control. The WB bands intensity of representative gels was quantified by densitometry and normalized to GAPDH. The densitometry values represent the mean (% of control) ± SD of three experiments. Statistical significances were expressed as *$P < 0.05$; vs. control cells (0 nM of PEPD, without IL-1β).

1.5. AKT expression

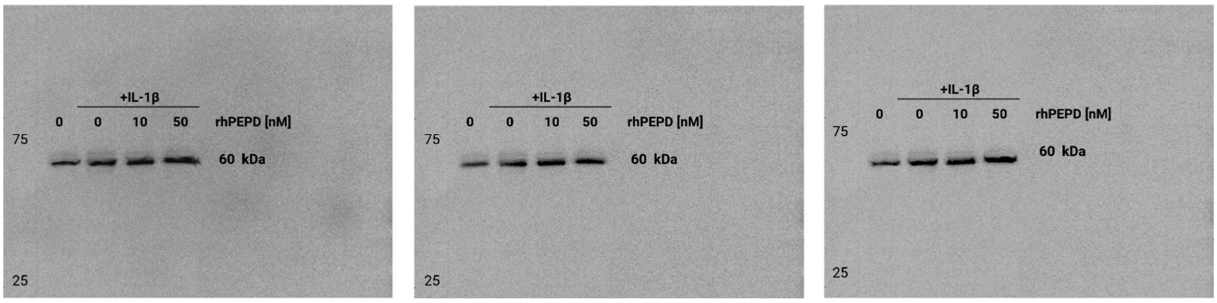

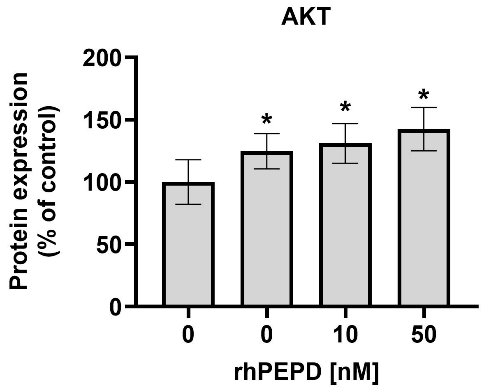

Figure S5. The AKT expressions in rhPEPD-treated fibroblasts (rhPEPD, 10, 50 nM) for 24 h in presence or absence of IL-1β (10ng/ml). GAPDH expression was used as a loading control. The WB bands intensity of representative gels was quantified by densitometry and normalized to GAPDH. The densitometry values represent the mean (% of control) ± SD of three experiments. Statistical significances were expressed as *$P < 0.05$; vs. control cells (0 nM of PEPD, without IL-1β).

1.6. Phospho-AKT expression

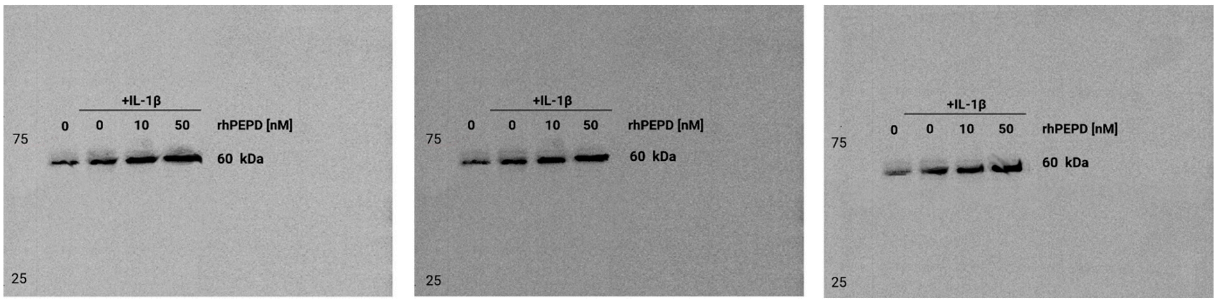

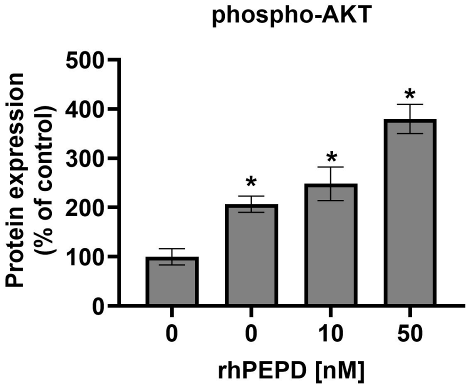

Figure S6. The phospho-AKT expressions in rhPEPD-treated fibroblasts (rhPEPD, 10, 50 nM) for 40 min in presence or absence of IL-1β (10ng/ml). GAPDH expression was used as a loading control. The WB bands intensity of representative gels was quantified by densitometry and normalized to GAPDH. The densitometry values represent the mean (% of control) ± SD of three experiments. Statistical significances were expressed as *$P < 0.05$; vs. control cells (0 nM of PEPD, without IL-1β).

1.7. mTOR expression

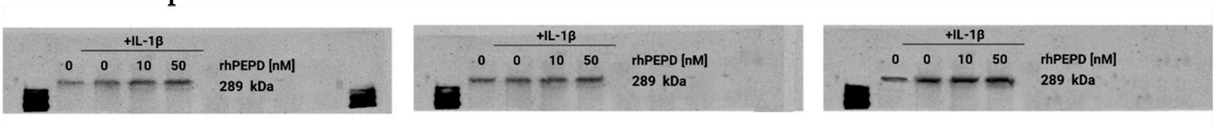

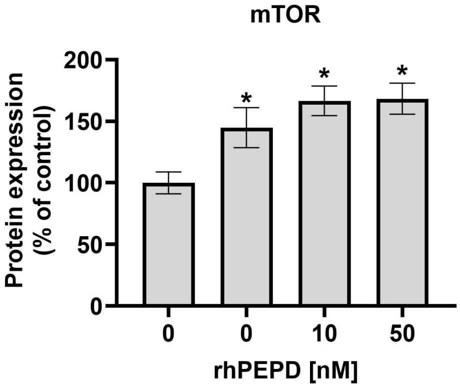

Figure S7. The mTOR expressions in rhPEPD-treated fibroblasts (rhPEPD, 10, 50 nM) for 24 h in presence or absence of IL-1β (10ng/ml). GAPDH expression was used as a loading control. The WB bands intensity of representative gels was quantified by densitometry and normalized to GAPDH. The densitometry values represent the mean (% of control) ± SD of three experiments. Statistical significances were expressed as *$P < 0.05$; vs. control cells (0 nM of PEPD, without IL-1β).

1.8. Phospho-mTOR expression

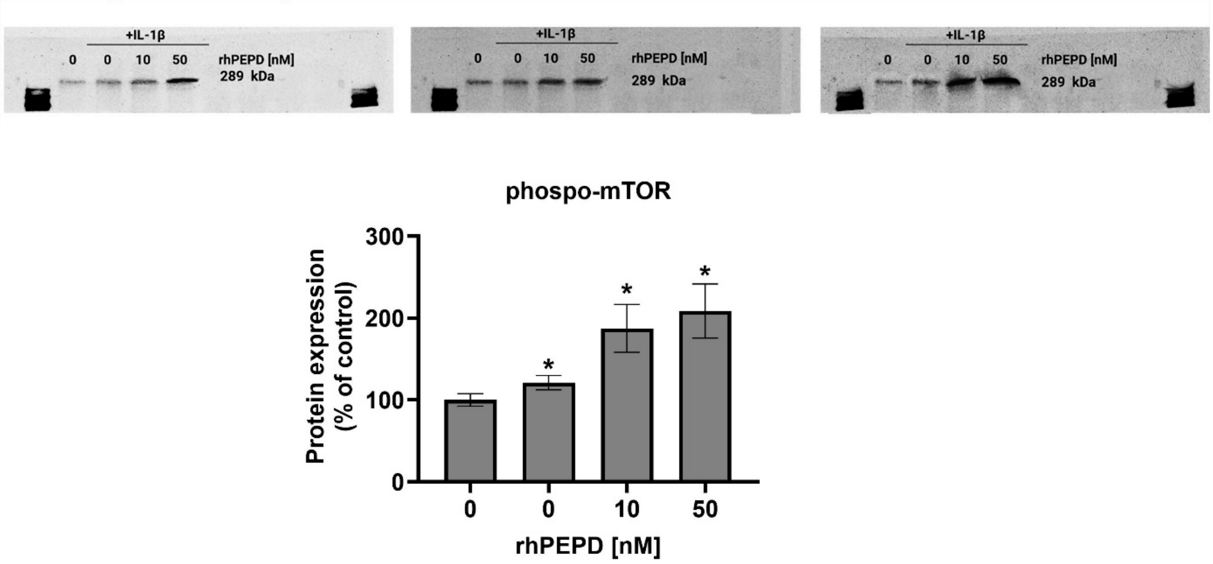

Figure S8. The phospho-mTOR expressions in rhPEPD-treated fibroblasts (rhPEPD, 10, 50 nM) for 40 min in presence or absence of IL-1β (10ng/ml). GAPDH expression was used as a loading control. The WB bands intensity of representative gels was quantified by densitometry and normalized to GAPDH. The densitometry values represent the mean (% of control) ± SD of three experiments. Statistical significances were expressed as *$P < 0.05$; vs. control cells (0 nM of PEPD, without IL-1β).

1.9. GAPDH expression

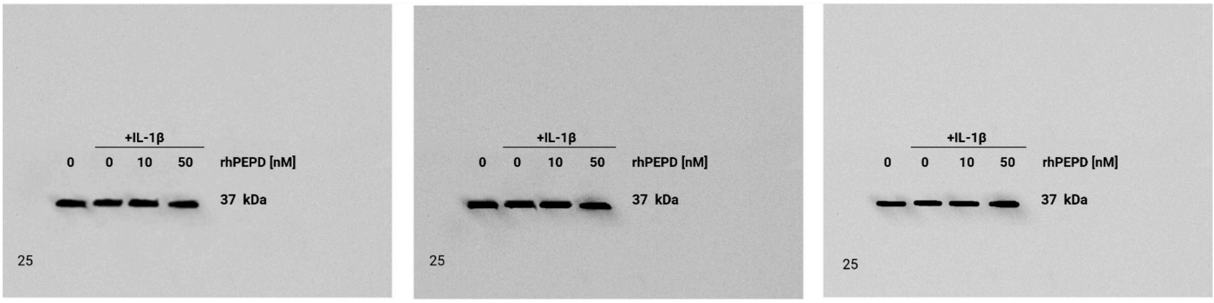

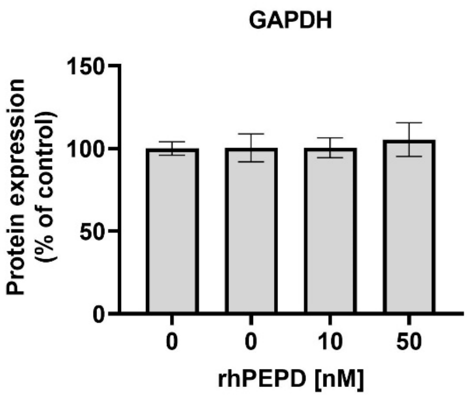

Figure S9. The GAPDH expressions in rhPEPD-treated fibroblasts (rhPEPD, 10, 50 nM) 24 h in presence or absence of IL-1β (10ng/ml). GAPDH expression was used as a loading control. The WB bands intensity of representative gels was quantified by densitometry and normalized to GAPDH. The densitometry values represent the mean (% of control) ± SD of three experiments. Statistical significances were expressed as *$P < 0.05$; vs. control cells (0 nM of PEPD, without IL-1β).

2. Representative blots from Western blotting analysis presented in Figure 3B.

2.1. EGFR expression

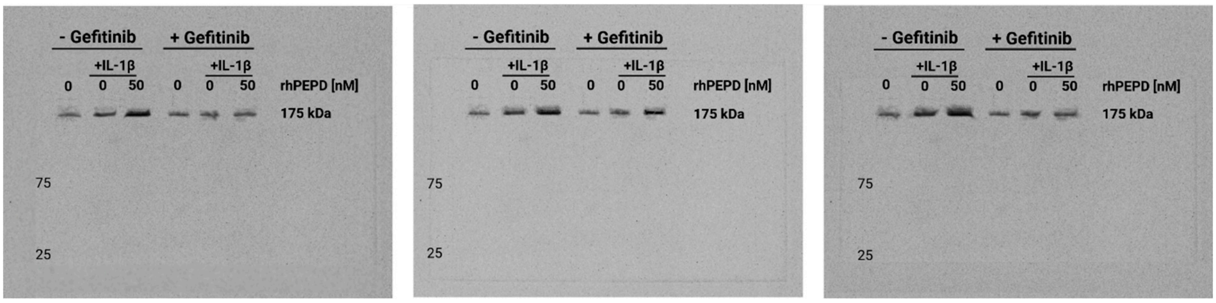

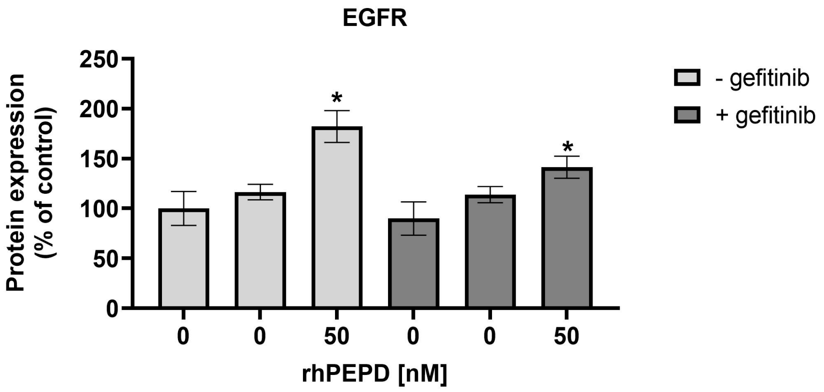

Figure S10. The EGFR expressions in lysates of rhPEPD-treated fibroblasts (rhPEPD, 50 nM) and pretreated with an inhibitor of EGFR (gefitinib, 45 µM for 2 h) cultured for 24 h in presence or absence IL-1β. (10ng/ml). GAPDH expression was used as a loading control. The WB bands intensity of representative gels was quantified by densitometry and normalized to GAPDH. The

densitometry values represent the mean (% of control) ± SD of three experiments. Statistical significances were expressed as *$P < 0.05$; vs. control cells (0 nM of PEPD, without IL-1β).

2.2. Phospho-EGFR expression

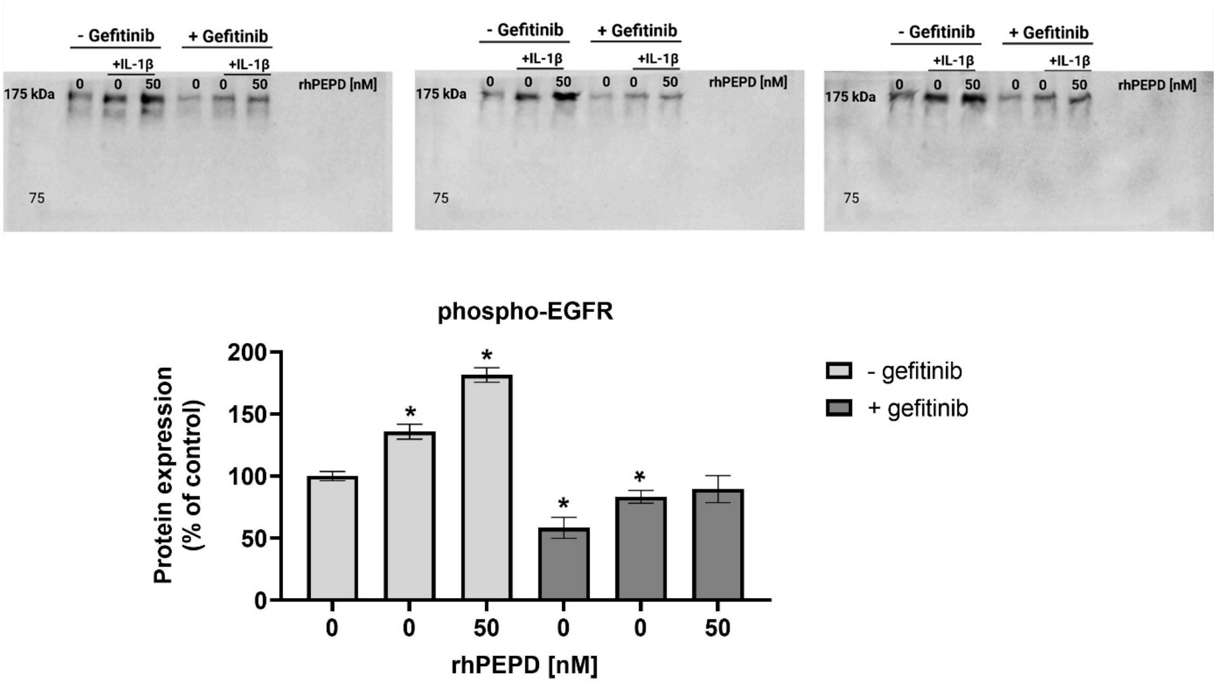

Figure S11. The phospho-EGFR expressions in lysates of rhPEPD-treated fibroblasts (rhPEPD, 50 nM) and pretreated with an inhibitor of EGFR (gefitinib, 45 μM for 2 h) cultured for 40 min in presence or absence IL-1β. (10ng/ml). GAPDH expression was used as a loading control. The WB bands intensity of representative gels was quantified by densitometry and normalized to GAPDH. The densitometry values represent the mean (% of control) ± SD of three experiments. Statistical significances were expressed as *$P < 0.05$; vs. control cells (0 nM of PEPD, without IL-1β).

Ratio of phospho-EGFR/EGFR expression

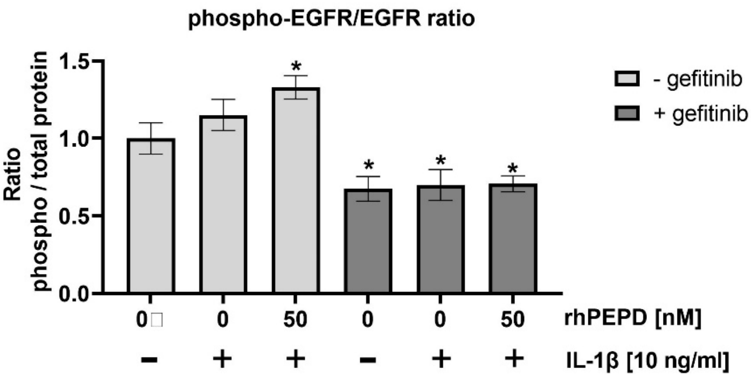

2.3. PI3K expression

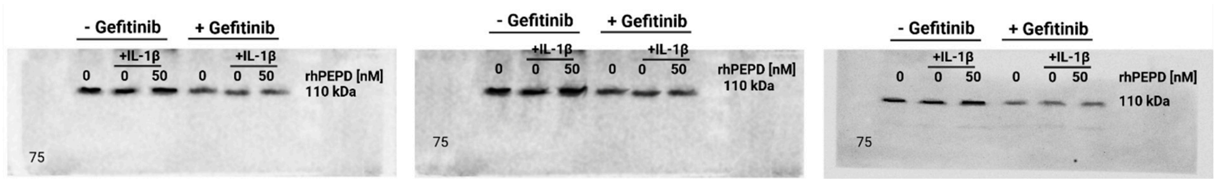

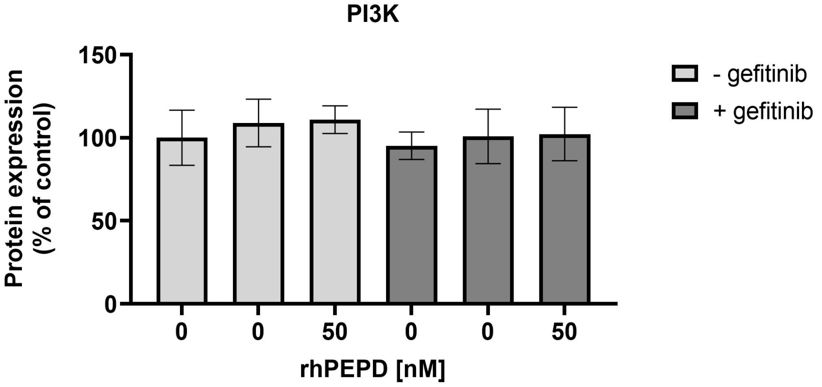

Figure S12. The PI3K expressions in lysates of rhPEPD-treated fibroblasts (rhPEPD, 50 nM) and pretreated with an inhibitor of EGFR (gefitinib, 45 μM for 2 h) cultured for 24 h in presence or absence IL-1β. (10ng/ml). GAPDH expression was used as a loading control. The WB bands intensity of representative gels was quantified by densitometry and normalized to GAPDH. The densitometry values represent the mean (% of control) ± SD of three experiments. Statistical significances were expressed as *$P < 0.05$; vs. control cells (0 nM of PEPD, without IL-1β).

2.4. Phospho-PI3K expression

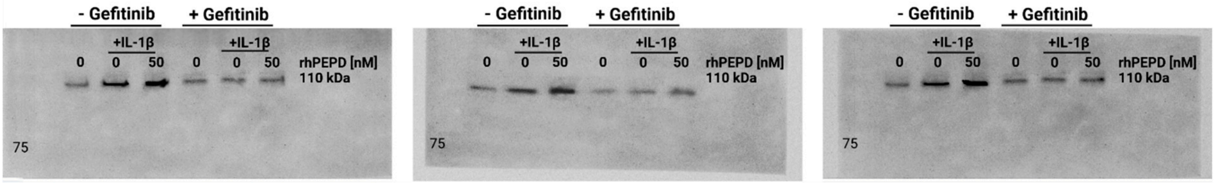

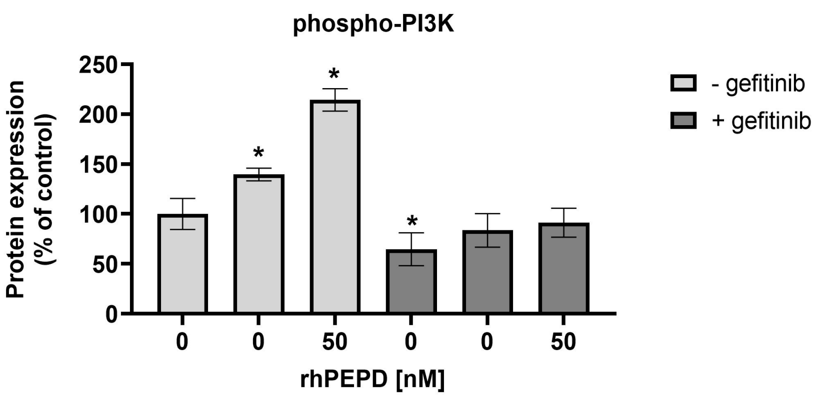

Figure S13. The phospho-PI3K expressions in lysates of rhPEPD-treated fibroblasts (rhPEPD, 50 nM) and pretreated with an inhibitor of EGFR (gefitinib, 45 μM for 2 h) cultured for 40 min in presence or absence IL-1β. (10ng/ml). GAPDH expression was used as a loading control. The WB bands intensity of representative gels was quantified by densitometry and normalized to GAPDH. The densitometry values represent the mean (% of control) ± SD of three experiments. Statistical significances were expressed as *$P < 0.05$; vs. control cells (0 nM of PEPD, without IL-1β).

Ratio of phospho-PI3K/PI3K expression

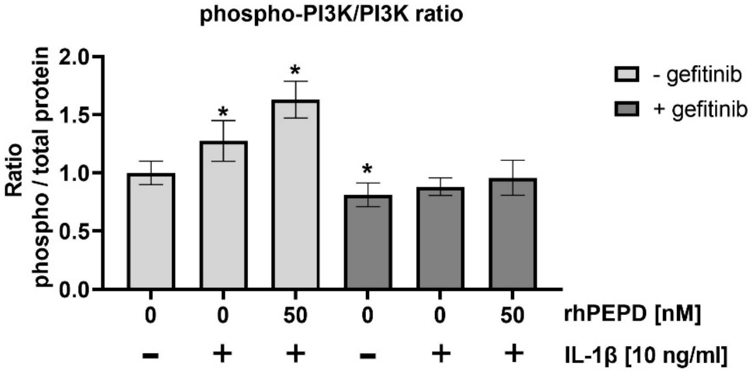

2.5. AKT expression

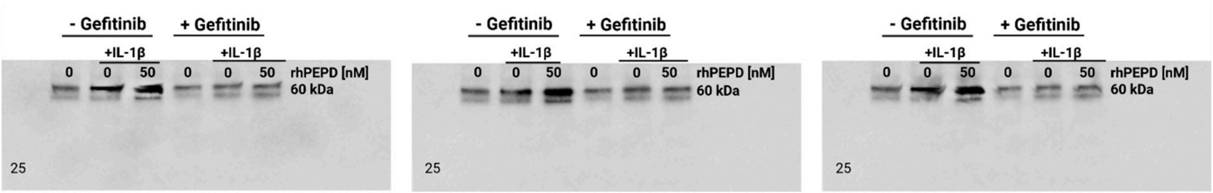

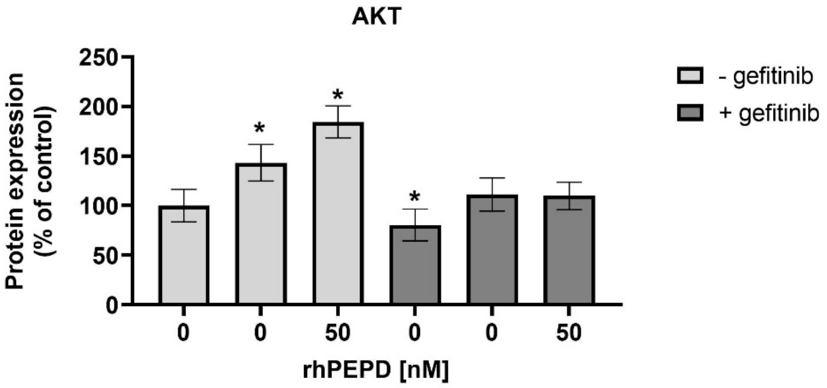

Figure S14. The AKT expressions in lysates of rhPEPD-treated fibroblasts (rhPEPD, 50 nM) and pretreated with an inhibitor of EGFR (gefitinib, 45 μM for 2 h) cultured for 24 h in presence or absence IL-1β. (10ng/ml). GAPDH expression was used as a loading control. The WB bands intensity of representative gels was quantified by densitometry and normalized to GAPDH. The densitometry values represent the mean (% of control) ± SD of three experiments. Statistical significances were expressed as *$P < 0.05$; vs. control cells (0 nM of PEPD, without IL-1β).

2.6. Phospho-AKT expression

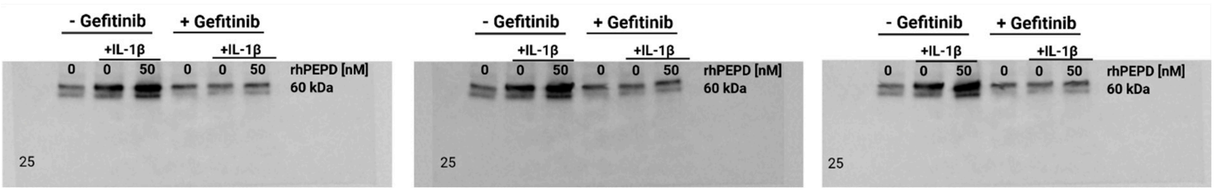

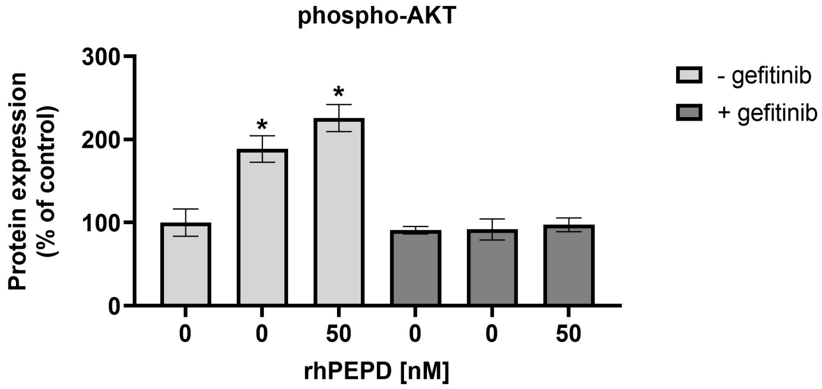

Figure S15. The phospho-AKT expressions in lysates of rhPEPD-treated fibroblasts (rhPEPD, 50 nM) and pretreated with an inhibitor of EGFR (gefitinib, 45 μM for 2 h) cultured for 40 min in presence or absence IL-1β. (10ng/ml). GAPDH expression was used as a loading control. The WB bands intensity of representative gels was quantified by densitometry and normalized to GAPDH. The densitometry values represent the mean (% of control) ± SD of three experiments. Statistical significances were expressed as *$P < 0.05$; vs. control cells (0 nM of PEPD, without IL-1β).

Ratio of phospho-AKT/AKT expression

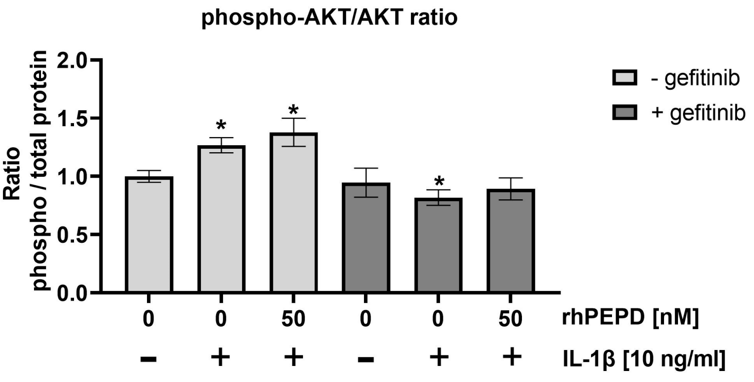

2.7. mTOR expression

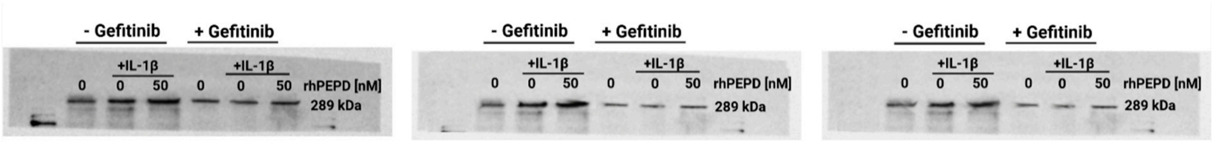

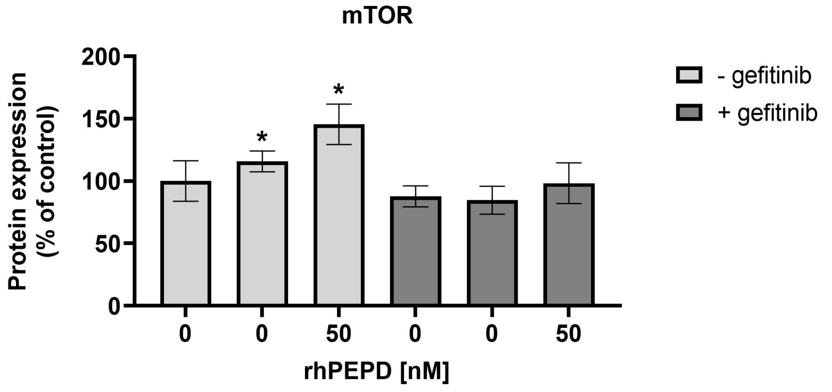

Figure S16. The mTOR expressions in lysates of rhPEPD-treated fibroblasts (rhPEPD, 50 nM) and pretreated with an inhibitor of EGFR (gefitinib, 45 µM for 2 h) cultured for 24 h in presence or absence IL-1β. (10ng/ml). GAPDH expression was used as a loading control. The WB bands intensity of representative gels was quantified by densitometry and normalized to GAPDH. The densitometry values represent the mean (% of control) ± SD of three experiments. Statistical significances were expressed as *$P < 0.05$; vs. control cells (0 nM of PEPD, without IL-1β).

2.8. Phospho-mTOR expression

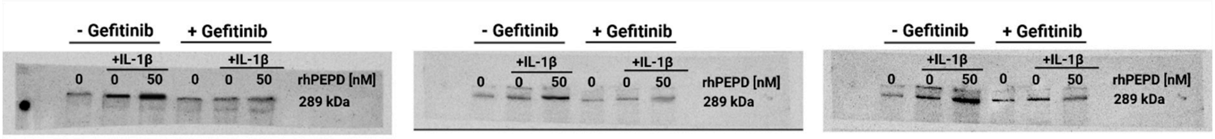

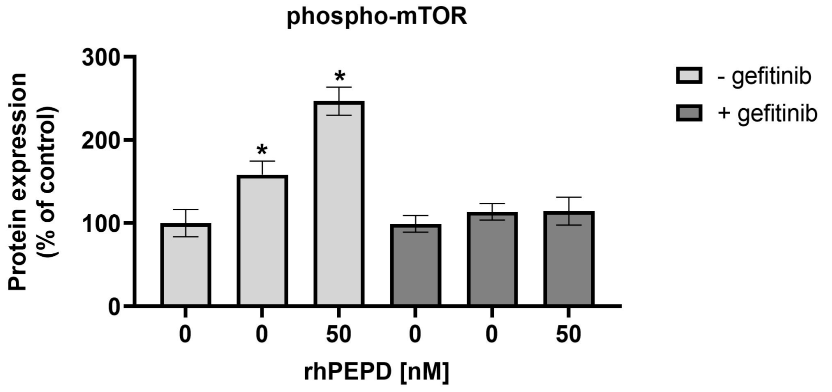

Figure S17. The phospho-mTOR expressions in lysates of rhPEPD-treated fibroblasts (rhPEPD, 50 nM) and pretreated with an inhibitor of EGFR (gefitinib, 45 µM for 2 h) cultured for 40 min in presence or absence IL-1β. (10ng/ml). GAPDH expression was used as a loading control. The WB bands intensity of representative gels was quantified by densitometry and normalized to GAPDH. The densitometry values represent the mean (% of control) ± SD of three experiments. Statistical significances were expressed as *$P < 0.05$; vs. control cells (0 nM of PEPD, without IL-1β).

Ratio of phospho-mTOR/mTOR expression

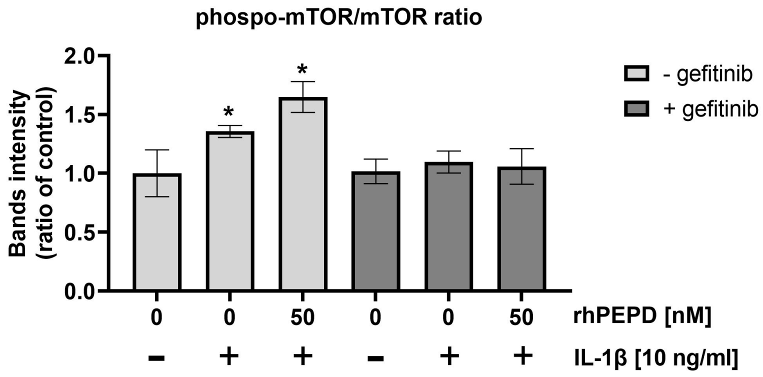

2.9. GAPDH expression

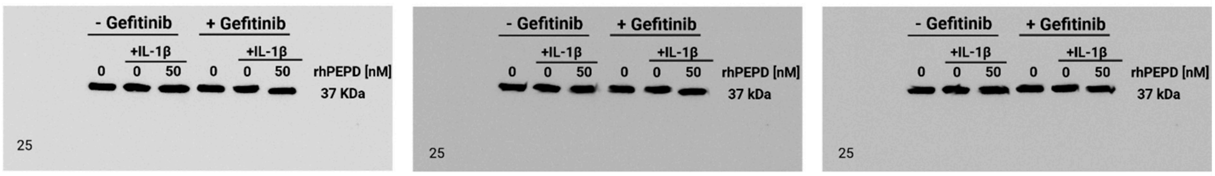

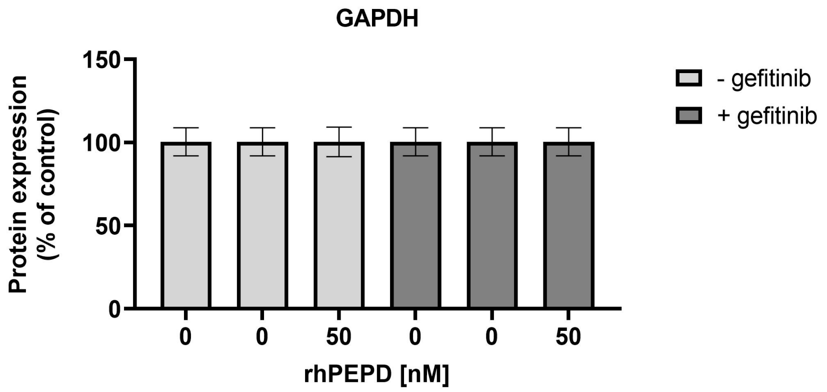

Figure S18. The GAPDH expressions in lysates of rhPEPD-treated fibroblasts (rhPEPD, 50 nM) and pretreated with an inhibitor of EGFR (gefitinib, 45 µM for 2 h) cultured for 24 h in presence or absence IL-1β. (10ng/ml). GAPDH expression was used as a loading control. The WB bands intensity of representative gels was quantified by densitometry and normalized to GAPDH. The densitometry values represent the mean (% of control) ± SD of three experiments. Statistical significances were expressed as $*P < 0.05$; vs. control cells (0 nM of PEPD, without IL-1β).

3. Representative blots from Western blotting analysis presented in Figure 4A.

3.1. β1 integrin expression

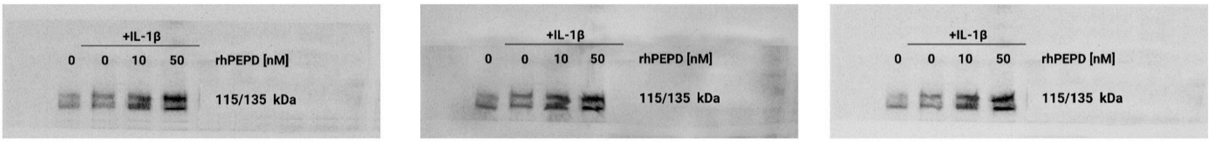

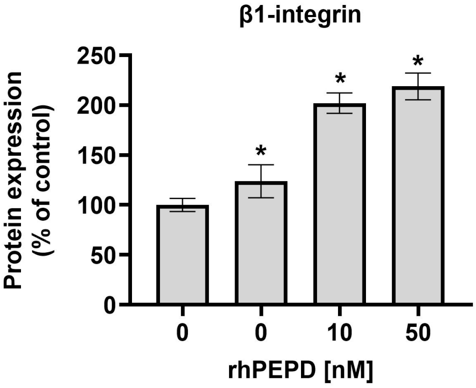

Figure S19. The β1 integrin expressions in rhPEPD-treated fibroblasts (rhPEPD, 10, 50 nM) for 24 h in presence or absence of IL-1β (10ng/ml). GAPDH expression was used as a loading control. The WB bands intensity of representative gels was quantified by densitometry and normalized to GAPDH. The densitometry values represent the mean (% of control) ± SD of three experiments. Statistical significances were expressed as $*P < 0.05$; vs. control cells (0 nM of PEPD, without IL-1β).

3.2. FAK expression

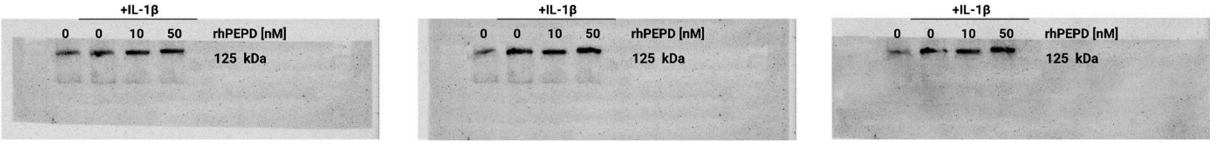

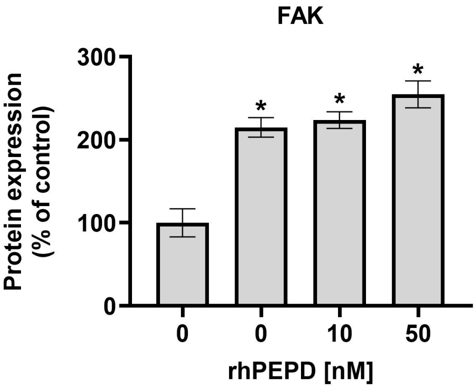

Figure S20. The FAK expressions in rhPEPD-treated fibroblasts (rhPEPD, 10, 50 nM) for 24 h in presence or absence of IL-1β (10ng/ml). GAPDH expression was used as a loading control. The WB bands intensity of representative gels was quantified by densitometry and normalized to GAPDH. The densitometry values represent the mean (% of control) ± SD of three experiments. Statistical significances were expressed as $*P < 0.05$; vs. control cells (0 nM of PEPD, without IL-1β).

3.3. Phospho-FAK expression

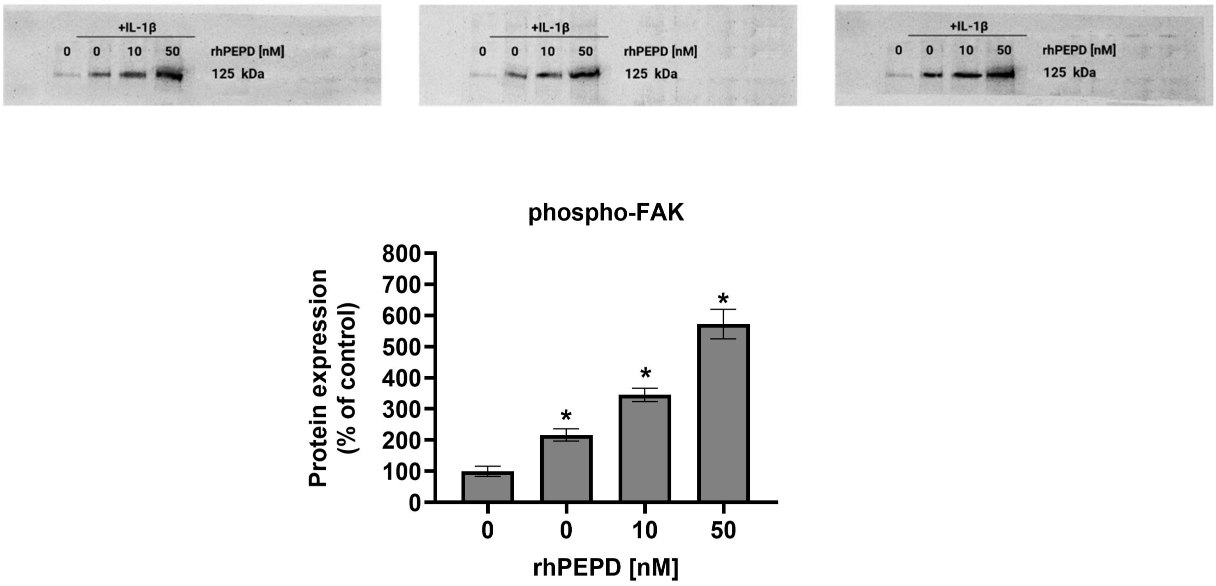

Figure S21. The phospho-FAK expressions in rhPEPD-treated fibroblasts (rhPEPD, 10, 50 nM) for 40 min in presence or absence of IL-1β (10ng/ml). GAPDH expression was used as a loading control. The WB bands intensity of representative gels was quantified by densitometry and normalized to GAPDH. The densitometry values represent the mean (% of control) ± SD of three experiments. Statistical significances were expressed as $*P < 0.05$; vs. control cells (0 nM of PEPD, without IL-1β).

3.4. Grb2 expression

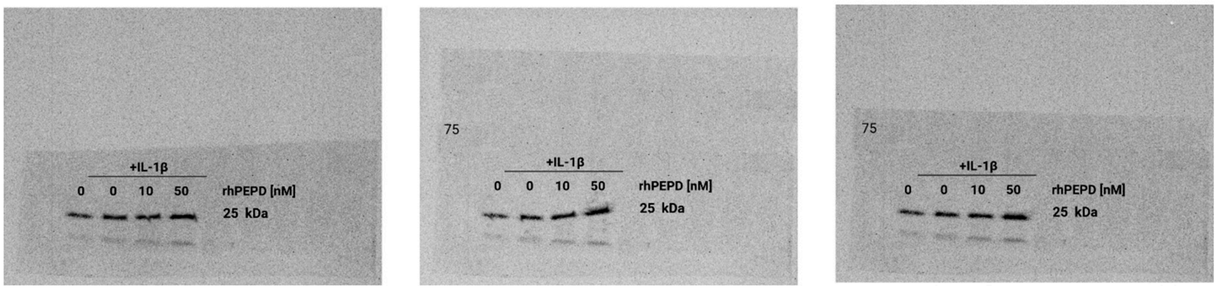

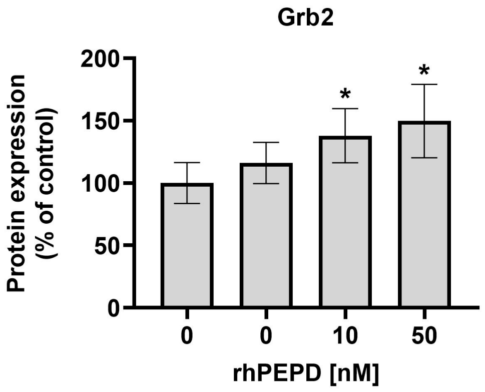

Figure S22. The Grb2 expressions in rhPEPD-treated fibroblasts (rhPEPD, 10, 50 nM) for 24 h in presence or absence of IL-1β (10ng/ml). GAPDH expression was used as a loading control. The WB bands intensity of representative gels was quantified by densitometry and normalized to GAPDH. The densitometry values represent the mean (% of control) ± SD of three experiments. Statistical significances were expressed as *$P < 0.05$; vs. control cells (0 nM of PEPD, without IL-1β).

3.5. ERK 1/2 expression

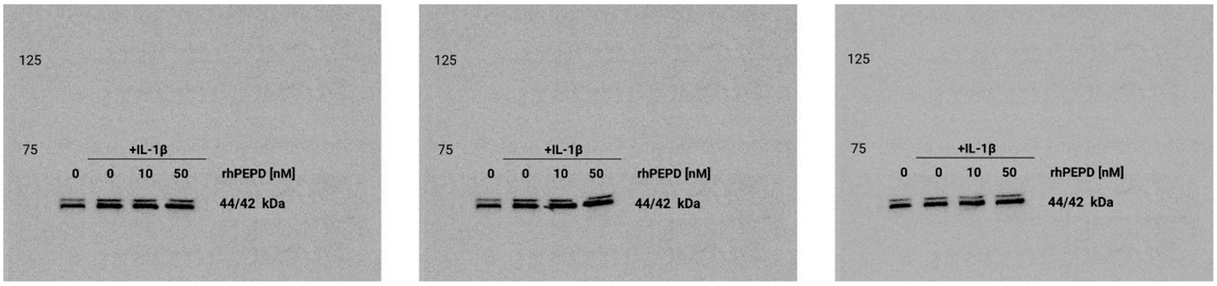

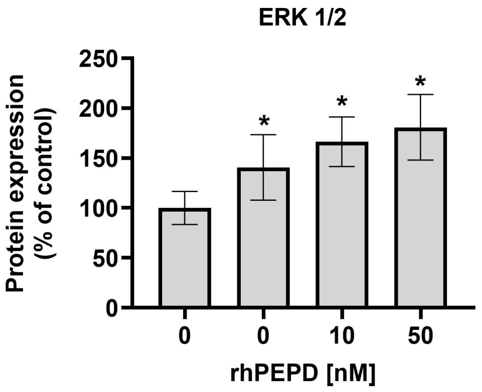

Figure S23. The ERK 1/2 expressions in rhPEPD-treated fibroblasts (rhPEPD, 10, 50 nM) for 40 min and 24 h in presence or absence of IL-1β (10ng/ml). GAPDH expression was used as a loading control. The WB bands intensity of representative gels was quantified by densitometry and normalized to GAPDH. The densitometry values represent the mean (% of control) ± SD of three experiments. Statistical significances were expressed as *$P < 0.05$; vs. control cells (0 nM of PEPD, without IL-1β).

3.6. Phospho-ERK 1/2 expression

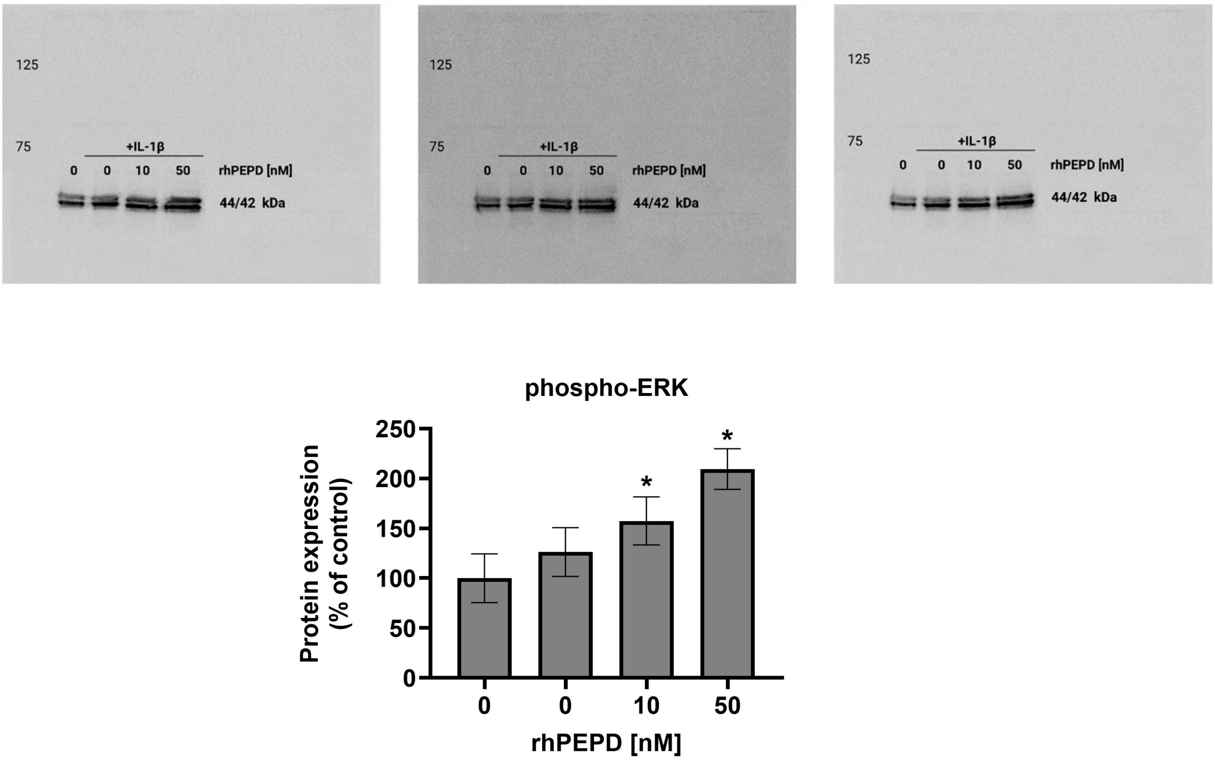

Figure S24. The phosphor-ERK 1/2 expressions in rhPEPD-treated fibroblasts (rhPEPD, 10, 50 nM) for 40 min in presence or absence of IL-1β (10ng/ml). GAPDH expression was used as a loading control. The WB bands intensity of representative gels was quantified by densitometry and normalized to GAPDH. The densitometry values represent the mean (% of control) ± SD of three experiments. Statistical significances were expressed as *$P < 0.05$; vs. control cells (0 nM of PEPD, without IL-1β).

4. Representative blots from Western blotting analysis presented in Figure 4B.

4.1. β1 integrin expression

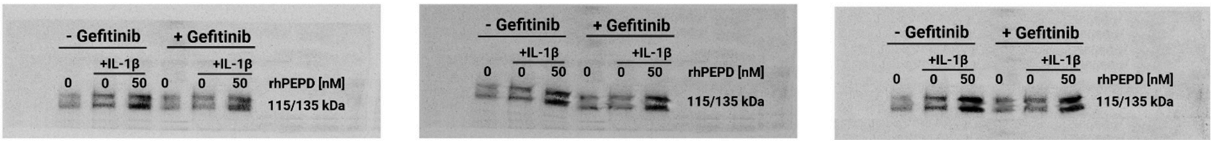

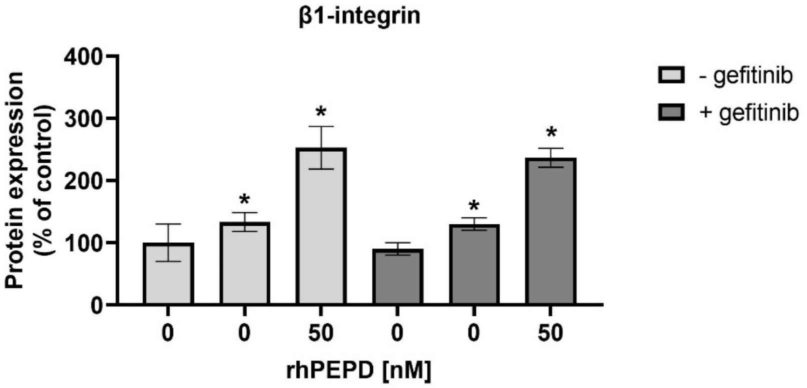

Figure S25. B1 integrin expressions in lysates of rhPEPD-treated fibroblasts (rhPEPD, 50 nM) and pretreated with an inhibitor of EGFR (gefitinib, 45 μM for 2 h) cultured for 24 h in presence or absence IL-1β. (10ng/ml). GAPDH expression was used as a loading control. The WB bands intensity of representative gels was quantified by densitometry and normalized to GAPDH. The densitometry values represent the mean (% of control) ± SD of three experiments. Statistical significances were expressed as *$P < 0.05$; vs. control cells (0 nM of PEPD, without IL-1β).

4.2. Phospho-FAK expression

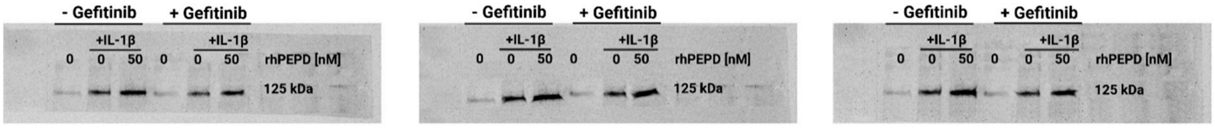

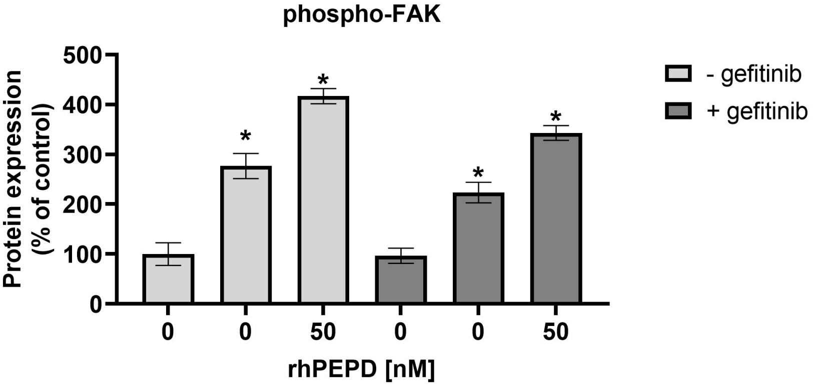

Figure S26. Phospho-FAK expressions in lysates of rhPEPD-treated fibroblasts (rhPEPD, 50 nM) and pretreated with an inhibitor of EGFR (gefitinib, 45 μM for 2 h) cultured for 40 min in presence or absence IL-1β. (10ng/ml). GAPDH expression was used as a loading control. The WB bands intensity of representative gels was quantified by densitometry and normalized to GAPDH. The densitometry values represent the mean (% of control) ± SD of three experiments. Statistical significances were expressed as *$P < 0.05$; vs. control cells (0 nM of PEPD, without IL-1β).

4.3. Grb2 expression

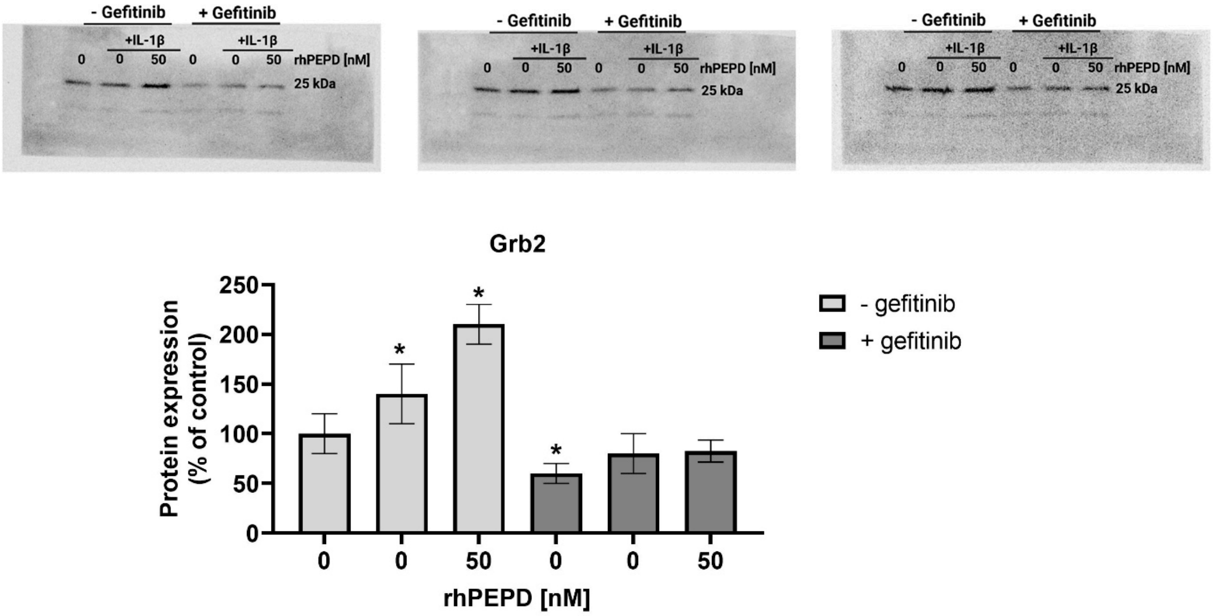

Figure S27. Grb2 expressions in lysates of rhPEPD-treated fibroblasts (rhPEPD, 50 nM) and pretreated with an inhibitor of EGFR (gefitinib, 45 μM for 2 h) cultured for 24 h in presence or absence IL-1β. (10ng/ml). GAPDH expression was used as a loading control. The WB bands intensity of representative gels was quantified by densitometry and normalized to GAPDH. The densitometry values represent the mean (% of control) ± SD of three experiments. Statistical significances were expressed as *$P < 0.05$; vs. control cells (0 nM of PEPD, without IL-1β).

4.4. ERK 1/2 expression

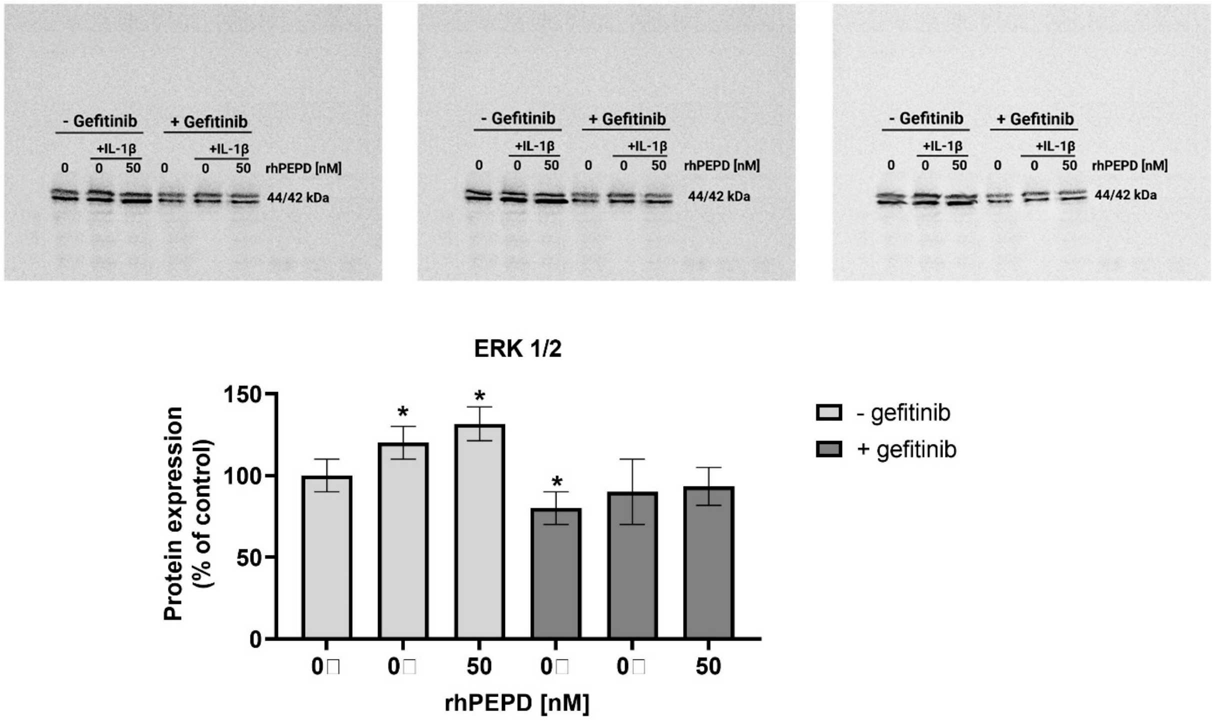

Figure S28. Erk1/2 expressions in lysates of rhPEPD-treated fibroblasts (rhPEPD, 50 nM) and pretreated with an inhibitor of EGFR (gefitinib, 45 μM for 2 h) cultured for 24 h in presence or absence IL-1β. (10ng/ml). GAPDH expression was used as a loading control. The WB bands intensity of representative gels was quantified by densitometry and normalized to GAPDH. The densitometry values represent the mean (% of control) ± SD of three experiments. Statistical significances were expressed as *$P < 0.05$; vs. control cells (0 nM of PEPD, without IL-1β).

4.5. Phospho-ERK 1/2 expression

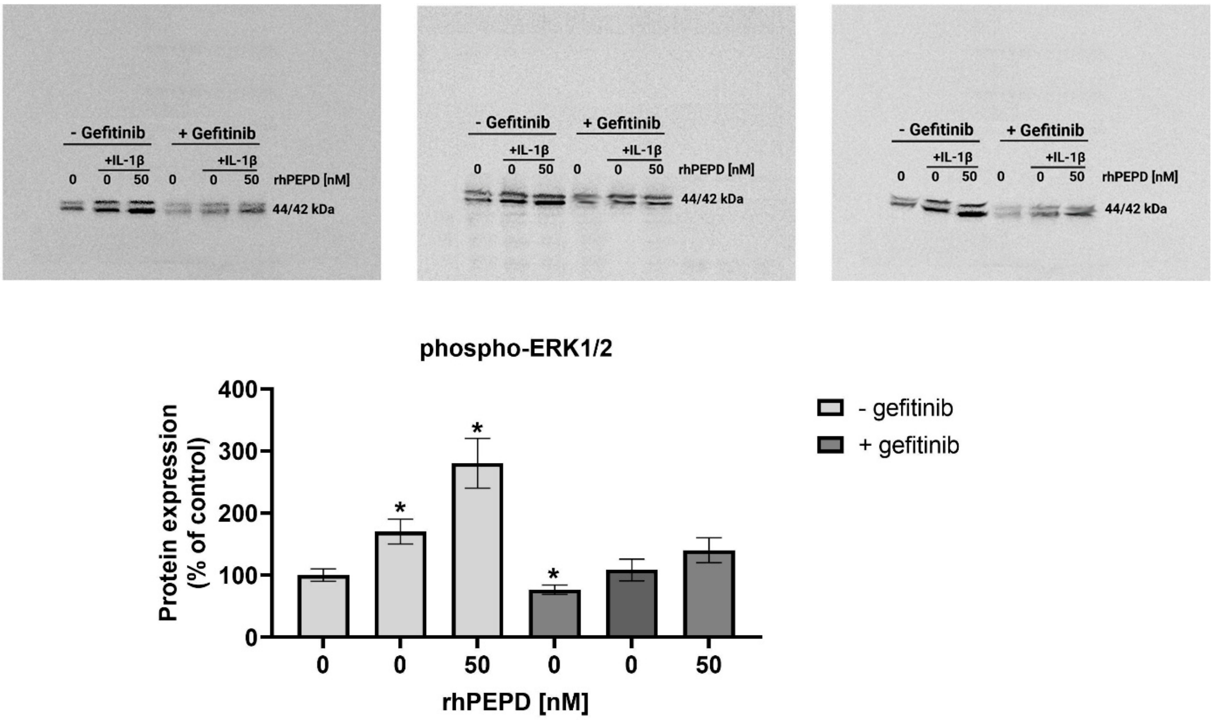

Figure S29. Phospho-Erk1/2 expressions in lysates of rhPEPD-treated fibroblasts (rhPEPD, 50 nM) and pretreated with an inhibitor of EGFR (gefitinib, 45 μM for 2 h) cultured for 40 min in presence or absence IL-1β. (10ng/ml). GAPDH expression was used as a loading control. The WB bands intensity of representative gels was quantified by densitometry and normalized to GAPDH. The densitometry values represent the mean (% of control) ± SD of three experiments. Statistical significances were expressed as *$P < 0.05$; vs. control cells (0 nM of PEPD, without IL-1β).

5. CellTiter-Blue Cell Viability Assay

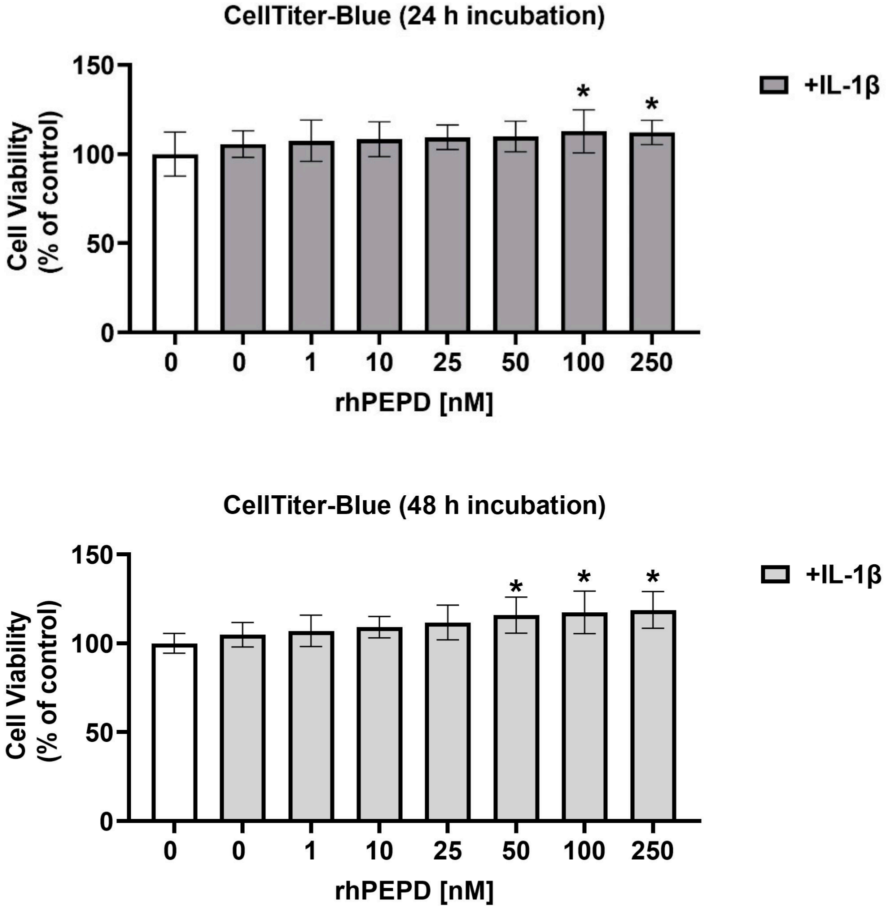

Figure S30. Cell viability of fibroblasts was measured using Cell Titer Blue assay as described in the manufacturer's protocol (Promega, Madison, WI, United States). Cells, seeded at 5 × 10^3 cells/well in a 96-well plate, were submitted to rhPEPD treatment at concentrations of 1–250 nM and IL-1*β* (10 ng/ml)-for 24 h and 48 h. Briefly, cells were incubated with a resazurin-containing solution at 37°C for 2 h. Absorbance was read on TECAN Infinite® M200 PRO (Tecan Group Ltd., Männedorf, Switzerland) at 570 and 600 nm as a reference wavelength. The results were presented as a percent of the control value. The mean values ± S.D. of three experiments done in duplicates are presented. The statistical significance was calculated vs. control (0 nM of rhPEPD, without IL-1β) and the results were considered significant at $^*P < 0.05$.

6. The influence of Gefitibin on the viability of fibroblasts

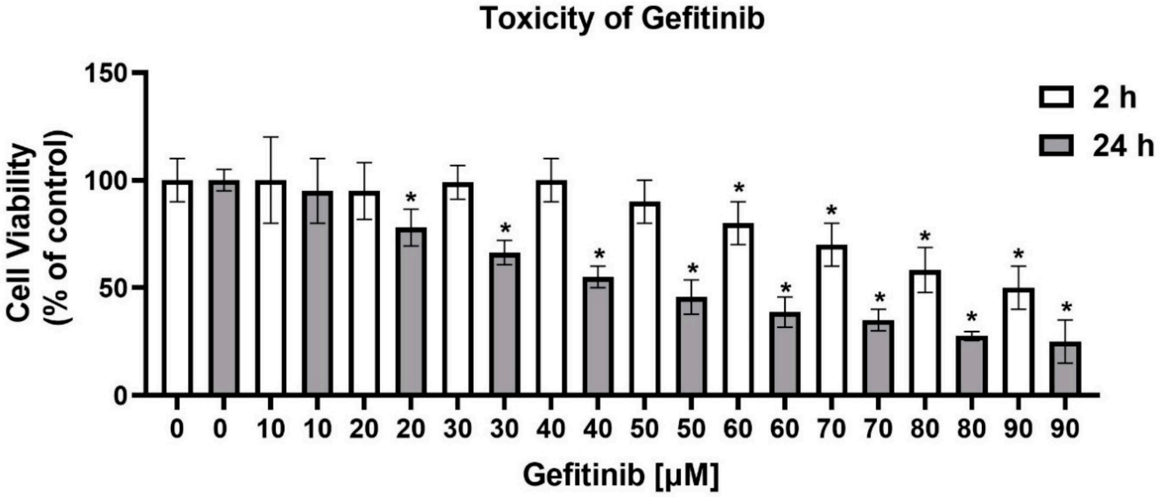

Figure S31. The influence of gefitinib on the viability of fibroblasts was measured using MTT test. Cells were treated of Gefitinib at concentrations of 0-90 μM for 2 h and 24 h. The results were presented as a percent of the control value. The mean values ± S.D. of three experiments done in duplicates are presented. The statistical significance was calculated vs. control (0 μM Gefitinib) and the results were considered significant at *P< 0.05.
